# Does Your Program Know Its AKI and CRRT Epidemiology? The Case for a Dashboard

**DOI:** 10.3389/fped.2020.00080

**Published:** 2020-03-06

**Authors:** Theresa A. Mottes

**Affiliations:** Texas Children's Hospital, Renal Section, Houston, TX, United States

**Keywords:** acute kidney injury (AKI), continuous renal replacement therapy (CRRT), quality improvement (QI), dashboard, standardization, epidemiology

## Abstract

Current acute kidney injury (AKI) literature focuses on diagnosis, treatment, and outcomes. While little literature exists studying the quality of care delivered to patients with AKI. However, improving outcomes for patients is dependent on the specifics of the delivered care (i.e., the who, what, when, and how). Therefore, it is necessary to direct attention to process measures to assess the relationship between care and outcomes. The application of quality improvement science to the care of AKI, uses a series of metrics encompassing both processes and outcomes to better understand, evaluate, and ensure the delivery high quality care.

## Introduction

Recent pediatric epidemiology and prevalence studies reported AKI developed in 26.9% of critically ill children, with severe AKI occurring 11.6% (1). The current management for AKI is support with interventions that target improving hemodynamics, removing the potential sources of the renal injury, and the utilization of renal replacement therapy in the setting of severe AKI (2). While diagnosis and treatment of AKI is well-described in the literature, very few studies focused on the quality of care delivered in the AKI care continuum. There is an assumption that care delivery is consistent between institutions, as well as within an institution. However, when examined further significant practice variations exist across the AKI care continuum (3, 4). Analyzing the relationship between process measures, outcomes measure, and patient outcomes is the foundation of quality improvement (QI) science. QI science performs a series of metrics encompassing both processes and outcomes to better understand, evaluate, and ensure the delivery high quality care. Process measures reflect adherence to the standardized steps and practices performed by health care staff members that are necessary to ensure quality care is delivered to every patient (5). Literature is skewed heavily toward understanding the associations between care and outcome measures (e.g., mortality). However, improving outcomes for patients is dependent on the specifics of the delivered care (i.e., the who, what, when, and how). More attention is now given to process measures, understanding how the degree of variability in the process of delivering care is associated with patient outcome.

Aimed at reducing practice variation and standardizing care for managing AKI, the 5Rs approach was recently introduced (6). The 5Rs approach identifies area for interventions along the AKI care continuum; Recognition, Response, Risk identification, Renal support, and Rehabilitation (6). Interventions in each category may include the use of electronic health record alerts to identify patients, provide clinical decision support algorithms for care delivery, implementation of care bundles to reduce variation, and development of a QI dashboard for reporting. Studying these process measures and the association with patient outcomes is the foundation for improving care.

## Standardize Care Delivery and QI Methodologies

QI practices are grounded in understanding how care delivery processes (and variation in processes) impact patient outcomes. Practice variation and its association with poor outcomes is well-described in the literature (7–9). For example, central line infections (outcome measure) are directly associated with mortality (patient outcome), so focus is given to measuring the performance of providers in actions aimed to reduce the infections such as bundles of care and infection prevention standards (process measures). This kind of quality improvement analysis is also evident in other complex therapies such as cardiopulmonary resuscitation—a reported process measure is depth of compressions in relation to published guidelines (10, 11). In these investigations, measuring process outcomes is leading to the understanding that the quality, consistency, and reproducibility of the care provided (and adherence to existing guidelines or benchmarks) is itself important. Therefore, the logical first step in applying QI strategies to an AKI/CRRT program is establishing standardized practices for the delivery of care. Standardization of practice is achieved by developing detailed recommendations to guide practitioners in providing appropriate evidence-based interventions [e.g., standard practice guidelines (SPG), care pathways, or care bundles].

The benefits of standardization are two-fold. Establishing a standard of care ensures each patient receives high quality consistent care, as well as providing a platform for standardizing team expectations and communication (12). For example, a SPG recommends performing a blood prime initiation procedure for patient weighing <10 kg. However, the orders are not consistent with the SPG. The team members recognize the variation in practice and communicates this with ordering practitioner, preventing an error and potential harm. It is important to acknowledge that SPG are meant to provide recommendations for care and do not limit the practitioner from using expertise to modify interventions or therapies based on patient responses.

Following the implementation of standardized practices, the next steps are data collection, data analysis, and preparing and distributing reports. An essential component of data analysis is identifying deviations from established benchmarks or goals. Upon the detection of a deviation, a “deep dive” is done to investigate for potential causes through factor analysis of patient (selection criteria, initiation, size and body habitus, special circumstances), equipment (inclusive of access catheter and machine), technical proficiency (nursing care and pharmacy), and the prescription of the therapy (modality, dose, anticoagulation) (2). Interventions, if necessary, are based on the final results of the “deep dive.”

## Quality Improvement in AKI

### Recognition (Early)/Response/Risk Identification

The early recognition of AKI has been associated with improved patient outcomes. Forde and colleagues report earlier diagnosis of AKI and improvement AKI management following targeted education and implementation of a checklist (13). The AKI checklist/bundle uses a simple acronym ABCDE; Address drugs, Boost blood pressure, Calculate fluid balance, Dip urine, and Exclude obstruction (13). The primary aim, diagnosing AKI within 24 h, improved from 30 to 100%. The checklist was implemented 75% of the time in the post-education period.

Other early recognition QI programs utilize the electronic health records to detect patient at risk for AKI or have AKI. The electronic health record identified the target population and alerts the practitioner. However, the literature suggests that alerting practitioners alone, does not improve patient outcomes (14). A systematic integration of clinical decision support with the alert is necessary to influence patient outcomes (15). The ideal AKI QI initiative aimed at recognition involves detected AKI, followed by an alert to the appropriate personnel and recommends intervention of preventative and therapeutic measures.

### Renal Support

As previously discussed, there is a paucity of literature studying the delivery of renal replacement therapy (RRT). The majority of the research is focused on patient characteristics, indications for RRT, and patient outcomes (16–18). The care is continuous in nature and involves numerous processes for safe, effective care to be delivered. Studying process measures, both categorical and temporal, provides an index of quality by providing a quantifiable level of adherence to accepted performance standards.

#### Activity

Activity metrics are tracked to study the relationship between frequency of therapies and other process measures. Specifically, assessing if available resources are sufficient to deliver high quality of care.

#### Filter Survival

The optimal delivery of CRRT is contingent on maintaining a well-functioning CRRT circuit. However, filter life is multifactorial and therefore is assessed using two process measures: filter life and unplanned filter changes (UPC).

##### Filter life

Filter life is defined as the duration of time, measured in hours, an individual filter or circuit is delivering therapy to the patient (2, 19).

##### Unplanned filter changes

UPC is defined as any filter changed prior to 60 h, censored for patient procedures, emergent events or patient death (2, 19).

##### Prescription

Prescribed and achieved CRRT effluent doses are an important process measures and provides an objective assessment of the delivered care. Variations between prescribed and delivered was quantified by simultaneously measuring both values and calculating % delivered (2, 19).

##### Minimum prescription

The minimum prescription is defined as the total number of CRRT hours and the total effluent measured in mL normalized to patient body surface area. The standard prescription for pediatrics is 2,000 ml/1.73 m^2^/h. Therefore, prescription below this dose were identified as a deviation from the standard.

##### Average treatment time

The average treatment time is the quantified average of time the CRRT delivered therapy for an individual patient treatment course on CRRT (2).

##### Fluid balance

Fluid as a metric, is separated into fluid status at initiation and achievement of daily fluid goals. Fluid accumulation is expressed as percent fluid overload (% FO). The formula for calculating fluid overload is: [((Intake (liters) from ICU admission to CRRT start – Output (liters) from ICU admission to CRRT start)/1000))/ICU admission weight (kg)] (20).

Achieved Fluid Goal (Desired Total Fluid Output) is defined as achieving the established fluid goal within the acceptable range of a fluid goal is ±10% of target. Calculation of the variability from the target fluid goal assumes the actual 24-h total output will be equivalent to the total 24-h intake minus the net 24-h fluid balance goal (2).

### Rehabilitation

The final R in the AKI continuum is Rehabilitation. Recent literature report patients who recover from an AKI event have an increase in risk for developing chronic kidney disease (CKD) (21, 22). Therefore, using QI strategies to ensure adequate follow-up for AKI survivors is essential. Currently, there is a lack of evidence that answers the questions regarding follow-up (e.g., who, what, when, and how). Recent literature reported the use of an algorithm for establishing follow-up standards and well as what patient measures to assess (22).

## Conclusion

The use of an AKI dashboard provides and ongoing assessment of process measures and facilitates analyses of variations and deviations from standards of care. Assumptions about how effective the therapy is cannot be made simply by whether a patient survives. Ultimately, process metrics are valuable to study in and of themselves but are likely directly impactful to the traditional hard patient outcomes specific to the kidney, to morbidity, and to mortality.

## Author Contributions

TM collected, analyzed and interpreted the data for quality improvement in CRRT, as well as a major contributor in writing the manuscript.

### Conflict of Interest

The author declares that the research was conducted in the absence of any commercial or financial relationships that could be construed as a potential conflict of interest.
